# Distinct difference in tumor-infiltrating immune cells between Wilms’ tumor gene 1 peptide vaccine and anti-programmed cell death-1 antibody therapies

**DOI:** 10.1093/noajnl/vdab091

**Published:** 2021-06-29

**Authors:** Chisato Yokota, Jun Nakata, Koji Takano, Hiroko Nakajima, Hiromu Hayashibara, Hikaru Minagawa, Yasuyoshi Chiba, Ryuichi Hirayama, Noriyuki Kijima, Manabu Kinoshita, Yoshiko Hashii, Akihiro Tsuboi, Yoshihiro Oka, Yusuke Oji, Atsushi Kumanogoh, Haruo Sugiyama, Naoki Kagawa, Haruhiko Kishima

**Affiliations:** 1 Department of Neurosurgery, Osaka University Graduate School of Medicine, Suita, Osaka, Japan; 2 Department of Clinical Laboratory and Biomedical Sciences, Osaka University Graduate School of Medicine, Suita, Osaka, Japan; 3 Department of Neurosurgery, Osaka International Cancer Institute, Osaka, Osaka, Japan; 4 Department of Cancer Immunology, Osaka University Graduate School of Medicine, Suita, Osaka, Japan; 5 Department of Pediatrics, Osaka University Graduate School of Medicine, Suita, Osaka, Japan; 6 Department of Neurosurgery, Osaka Women’s and Children’s Hospital, Osaka, Izumi, Japan; 7 Department of Cancer Immunotherapy, Osaka University Graduate School of Medicine, Suita, Osaka, Japan; 8 Department of Cancer Stem Cell Biology, Osaka University Graduate School of Medicine, Suita, Osaka, Japan; 9 Department of Respiratory Medicine and Clinical Immunology, Osaka University Graduate School of Medicine, Suita, Osaka, Japan; 10 Department of Immunopathology, WP1 Immunology Frontier Research Center, Osaka University, Suita, Osaka, Japan

**Keywords:** cancer vaccine, combination therapy, glioblastoma, immunotherapy, PD-1, WT1

## Abstract

**Background:**

Wilms’ tumor gene 1 (WT1) peptide vaccine and anti-programmed cell death-1 (anti-PD-1) antibody are expected as immunotherapies to improve the clinical outcome of glioblastoma. The aims of this study were to clarify how each immunotherapy affects tumor-infiltrating immune cells (TIIs) and to determine whether the combination of these two therapies could synergistically work.

**Methods:**

Mice were transplanted with WT1 and programmed cell death-ligand 1 doubly expressing glioblastoma cells into brain followed by treatment with WT1 peptide vaccine, anti-PD-1 antibody, or the combination of the two, and survival of each therapy was compared. CD45^+^ cells were positively selected as TIIs from the brains with tumors, and TIIs were compared between WT1 peptide vaccine and anti-PD-1 antibody therapies.

**Results:**

Most mice seemed to be cured by the combination therapy with WT1 peptide vaccine and anti-PD-1 antibody, which was much better survival than each monotherapy. A large number of CD4^+^ T cells, CD8^+^ T cells, and NK cells including WT1-specific CD8^+^ and CD4^+^ T cells infiltrated into the glioblastoma in WT1 peptide vaccine-treated mice. On the other hand, the number of TIIs did not increase, but instead PD-1 molecule expression was decreased on the majority of the tumor-infiltrating CD8^+^ T cells in the anti-PD-1 antibody-treated mice.

**Conclusion:**

Our results clearly demonstrated that WT1 peptide vaccine and anti-PD-1 antibody therapies worked in the different steps of cancer-immunity cycle and that the combination of the two therapies could work synergistically against glioblastoma.

**Key Points**

- We established a mouse glioblastoma model for evaluation of WT1 vaccine and anti-PD-1 therapies.
- Analysis of TIIs showed that each therapy worked in different steps of cancer-immunity cycle.
- Combination therapy showed much better survivals than each monotherapy.

**Importance of the Study**

Both WT1 peptide vaccine and anti-PD-1 antibody are expected as immunotherapies against glioblastoma, and many clinical studies have been performed. We analyzed how each immunotherapy affect tumor-infiltrating immune cells (TIIs) by using a mouse glioblastoma model. A large number of CD4^+^ T cells, CD8^+^ T cells, and NK cells including WT1-specific T cells infiltrated into glioblastoma by WT1 peptide vaccine therapy. On the other hand, anti-PD-1 antibody therapy did not increase the infiltration of TIIs but induced low expression of PD-1 molecules on tumor-infiltrating CD8^+^ T cells. There results clearly demonstrated for the first time the striking difference in TIIs between the two therapies under the same tumor microenvironment. Since each therapy works in different steps of cancer-immunity cycle, their combination works synergistically. Consist with the finding, the combination therapy showed much better survival than each monotherapy in our mouse glioblastoma model. Our findings will lead to human clinical trials.

Glioblastoma is the most common and aggressive primary brain tumor in adults.^1^ Standard treatment for glioblastoma is surgical resection and postoperative chemo-radiotherapy. However, few patients can be cured by these standard treatments, and median survival time was reported to be less than 12 months.^2–5^ Therefore, new strategies that can improve such unsatisfactory prognosis are desired, and immunotherapy is one of the most attractive treatments to improve the bad prognosis.^6,7^

During the past decade, many types of immunotherapies such as monoclonal antibody, tumor-associated antigen (TAA) peptide vaccine, immune checkpoint inhibitor, and chimeric-antigen receptor T-cell therapies have been performed for various kinds of tumors. However, these immunotherapies have been clinically effective for only a fraction of patients. The accumulated knowledge about what kind of patients could have benefit from immunotherapy and how immunotherapy failed to show its efficacy induced a concept of a series of stepwise events, called the “cancer-immunity cycle,” which divided immune reaction from the initiation of immune cells to killing of tumor cells into seven steps.^8^ Based on this concept, the combination of several immunotherapies to cover the whole steps of cancer-immunity cycle is attracted attention to improve the efficacy of immunotherapy. Especially, the combination of TAA peptide vaccines and immune checkpoint inhibitors is expected to work synergistically to induce more effective anticancer immune response, since the former works as the accelerator of immune response and the latter as inhibitor for breaking system of immunity.^9–12^ To our knowledge, there are, however, no reports in which the whole steps of the cancer-immunity cycle were verified for these two immunotherapies under the same tumor microenvironment.

Wilms’ tumor gene 1 (WT1) is a TAA that was ranked as the top among 75 TAAs.^13,14^ Many doctor- and company-led clinical studies of the WT1 peptide vaccine have been performed with sufficient clinical efficacy, so that the WT1 peptide vaccine should be considered a promising immunotherapy.^15–17^ Anti-programmed cell death (anti-PD-1) antibody therapy, on the other hand, with a different anticancer activity from that of the WT1 peptide vaccine, is well established and widely used for various types of tumors.^18–21^ Therefore, the combination therapy of the WT1 peptide vaccine and the anti-PD-1 antibody is expected to induce stronger anticancer immunity compared to each therapy alone.

For this study, we established a mouse glioblastoma treatment model and clearly describe the different mechanisms in anticancer immunity between WT1 peptide vaccine and anti-PD-1 antibody therapies under the same tumor microenvironment as well as the functions of the two immunotherapies at each step of the cancer-immunity cycle.

## Materials and Methods

### Cells

GL261 murine glioblastoma cells were cultured and passaged in Dulbecco’s modified Eagle medium (DMEM; Sigma-Aldrich) containing 10% fetal bovine serum and 1% streptomycin. GL261 cells were transduced with mouse WT1 cDNA and then with Firefly Luciferase cDNA, and WT1-and Luciferase- doubly expressing GL261 cells, named GL261-WT1-luc, were established.

### Mice

B6N-Tyr^C-Brd^/BrdCrCrl (B6 Albino) male mice aged 6 weeks were purchased from Charles River Laboratories International Inc. All animal experiments in this study were approved by the Animal Experimentation Committee and Gene Modification Experiments Safety Committee of Osaka University (approval number: 28-078-008/4343) and were performed in accordance with the guidelines of the Animal Research Committee of the Osaka University Graduate School of Medicine.

### In Vivo Imaging System

Bioluminescence of transplanted tumors was measured by in vivo imaging system (IVIS; Xenogen). Luciferin (150 μg/g of mouse weight) was subcutaneously injected 30 minutes prior to imaging, and tumor-emitting photon counts were quantified using the Living Image 3.1 (Caliper Life Sciences).

### Transplantation of Glioblastoma Cells Into Brain

Mice were stereotactically injected with 3 × 10^6^ WT1-GL261-Luc cells in phosphate-buffered saline (PBS) of 3 μL into the right thalamus (2 mm lateral and 2 mm posterior from bregma, and 3 mm dorsoventral from dura) using a stereotaxic injector (KDS 310; Muromachi-kikai). Tumor engraftment was ensured by IVIS 5 days after the transplantation. Mice with tumor-emitting photon counts of 5.0 × 10^4^ or more had visible tumors at autopsy.

### Treatment of Tumor-Bearing Mice

For WT1 peptide vaccine therapy, both WT1_126–134_ (RMFPNAPYL) and WT1_35–52_ (WAPVLDFAPPGASAYGSL) peptides (SIGMA Genosys) were prepared as oil-in-water emulsion with Montanide ISA 51 (Seppic S.A.) and subcutaneously and weekly injected for a total of five times on axillary regions. Amino acid sequences of these two mouse WT1 peptides were the same as those of human WT1 peptides. For anti-PD-1 antibody therapy, 200 μg of anti-mouse PD-1 antibody (clone RMP1-14, BioXCell) was intraperitoneally injected twice a week for a total of nine times. In the combination therapy, WT1 peptide vaccine and anti-PD-1 antibody therapies were simultaneously performed.

### Hematoxylin and Eosin Staining and Immunohistochemistry

Tumor-bearing brains were fixed by vascular perfusion with 4% paraformaldehyde at 4°C and embedded in paraffin. Tissue sections of 5 μm were prepared and deparaffinized. For hematoxylin and eosin (HE) staining, sections were stained with hematoxylin for 8 minutes, following washing with PBS, and then with eosin for 2 minutes. For immunohistochemistry (IHC), after endogenous peroxidase-blocking with the 2% hydrogen peroxide in methanol for 10 minutes and washing in PBS, heat-mediated antigen retrieval was performed with Dako Target Retrieval Solution buffer (Ph6.0) (Dako Cytomation) at 120°C for 10 minutes. The sections were incubated with mouse anti-WT1 protein antibody (Abcam), or anti-programmed cell death-ligand 1 (anti-PD-L1) rabbit monoclonal antibody (Cell Signaling Technology) at 4°C overnight. WT1 and PD-L1 expression was visualized with the Vectastain ABC kit (Vector Laboratories) and diaminobenzidine (WAKO).

### Preparation of Tumor-Infiltrating Immune Cells

Analysis of TIIs was performed as previously reported.^22^ Mice with tumor-emitting photon counts of 5.0 × 10^4^ or more were sacrificed during from days 19 to 35 after transplantation of GL261-WT1-luc cells. The tumor-bearing hemispheres were resected and cut into small pieces in RPMI medium with enzymes of Tissue Dissociation Kit (Milteny Biotec), following mechanical gentle dissociation of cells by using gentle MACS Dissociator (Milteny Biotec). The cell suspension was filtered through a 100-µm nylon cell strainer (BD Biosciences), and then CD45.1^+^ cells were positively collected by using MagniSort Mouse CD45.1 Positive Selection Kit (Thermo Fisher Scientific) and used as TIIs.

### Flow Cytometry of TIIs

One part of the TIIs was stained with FITC anti-mouse CD3 antibody (17A2: Biolegend), PE anti-mouse CD4 (RM4-4: Biolegend), Alexa Fluor 647 CD8 (KT15: MBL), Brilliant Violet 510 anti-mouse/human CD11b (M1/70: Biolegend) and PE/Cy7 anti-mouse NK-1.1 (PK136: Biolegend). Another part of the TIIs was stained with H-2Db WT1 126–134 tetramer-RMFPNAPYL-PE (MBL), FITC anti-mouse CD3, Alexa Fluor 647 anti-mouse CD8, and Brilliant Violet 421 anti-mouse CD279 (PD-1) (RMP1-30: Biolegend). Since the isotype of anti-PD-1 antibody was a rat IgG2a, an anti-rat IgG2a antibody was used for the detection of binding of anti-PD-1 antibody on TIIs. TIIs were stained with FITC anti-mouse CD3, Alexa Fluor 647 anti-mouse CD8, and Brilliant Violet 421 anti-mouse CD279 (PD-1) antibodies (RMP1-30: Biolegend), washed three times with FACS buffer, and then stained with BV510 mouse anti-rat IgG1/IgG2a antibody (G28-5: BD Biosciences). Flow cytometry was performed on FACS Canto II or FACS Aria II (BD Biosciences).

For intracellular cytokine staining, CD4^+^ T cells were selectively collected from tumor cell suspension by using MagniSort Mouse CD4 Positive Selection Kit (Thermo Fisher Scientific), stimulated with WT1_35–52_ peptide, and then cultured in complete medium with 20 IU/mL interleukin-2 (Shionogi Biomedical Laboratories). Thirteen days later, the cultured CD4^+^ T cells were cocultured with splenocytes from a CD45.1 mouse pulsed or not pulsed with WT1_35–52_ peptide in complete medium containing with 10 μg/mL Brefeldin A (Sigma-Aldrich) for 4 hours, and stained with FITC anti-mouse CD3, APC-eFluor 780 anti-mouse CD4 (RM4-5: eBioscience), PE anti-mouse CD8α (53–6.7: eBioscience), Pacific Blue anti-mouse CD45.1 (A20: Biolegend), and Brilliant Violet 510 anti-mouse CD45.2 antibodies. Then, the cells were fixed, permeabilized by Cytofix/Cytoperm solution (BD Bioscience), and stained with PE/Cy7 anti-mouse interferon-γ (IFN-γ) (XMG1.2: Biolegend) and APC anti-mouse tumor necrosis factor-α (TNF-α) antibodies (MP6-XT22: Biolegend). Flow cytometry was performed on FACS Canto II (BD Biosciences).

### Statistical Analysis

All data obtained from the experiment were analyzed by JMP Pro software (version 14.0.0 SAS Institute). Survival curves were constructed using Kaplan-Meier methods and analyzed using the log-rank test. Wilcoxon’s test was used for the analysis of statistical difference in OS rates in each treatment.

## Results

### Establishment of a WT1 and PD-L1-Doubly Expressing Glioblastoma Mouse Model

**Figure 1.**
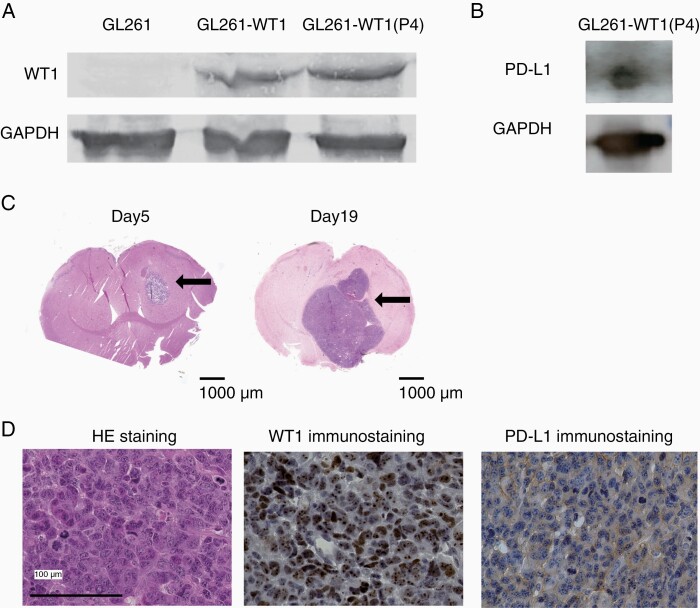
Establishment of PD-L1 and WT1 doubly expressing glioblastoma cells. (A) Western blotting analysis of WT1 protein for GL261 and GL261 transduced with WT1 cDNA (GL261-WT1). P4 means the GL261-WT1 cells that were four times passaged in culture. (B) Western blotting analysis of PD-L1 protein for GL261-WT1-luc. (C) Representative HE staining of brains on days 5 (left) and 19 (right) after the transplantation with GL261-WT1 glioblastoma cells. Intracranial tumor masses were indicated by arrows. (D) Representative HE and immunohistochemical staining of WT1 and PD-L1 proteins of resected tumors. WT1 and PD-L1 proteins were detected in the cytoplasm and on the cell surface, respectively.

GL261 cells, which are murine PD-L1 expressing astrocyte-derived tumor cells commonly used for mouse glioblastoma models, were transduced with mouse WT1 cDNA, and the continued WT1 protein expression of the resultant cells (GL261-WT1) was ensured by Western blotting analysis throughout many cell passages (Figure 1A). Next, the GL261-WT1 cells were transduced with Firefly Luciferase cDNA and PD-L1-, WT1-, and Luciferase- expressing GL261 cells, named GL261-WT1-luc, were established and stored in LN2 until use. Expression of PD-L1 protein of GL261-WT1-luc cells was ensured by Western blotting analysis (Figure 1B). GL261-WT1-luc cells that were stereotactically transplanted into the right thalamus of B6 Albino mice formed tumors on day 5 (Figure 1C). These tumors gradually progressed, spread around, and then caused extensive brain edema with midline shift on day 19 (Figure 1C). The mice showed weight loss and neurological symptoms such as hemiplegia, paraplegia, and general weakness and eventually fell into a coma. HE staining of the resected tumors confirmed that they had retained the original characteristics of glioblastoma (Figure 1D). Expression of both PD-L1 and WT1 proteins in the engrafted tumors was confirmed by immunohistochemical staining (Figure 1D). These results showed that the transplanted GL261-WT1-luc cells retained the original characteristics of WT1- and PD-L1-doubly expressing glioblastoma in the mouse brain.

### WT1 Peptide Vaccine and Anti-PD-1 Antibody Therapies in the Glioblastoma Mouse Model

**Figure 2.**
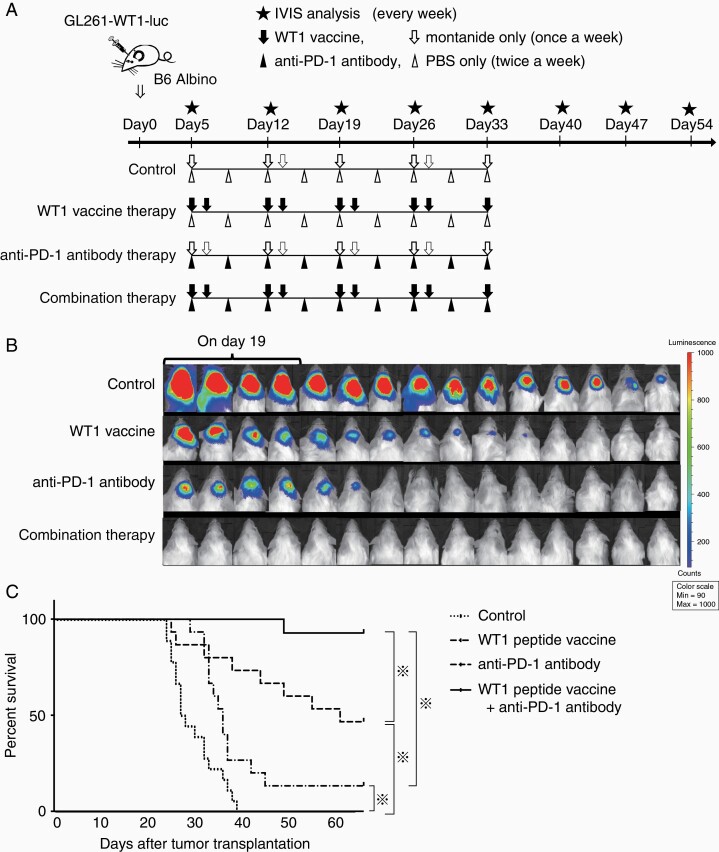
Treatment of glioblastoma-bearing mice with WT1 peptide vaccine, anti-PD-1 antibody, or the combination of the two. (A) A schema of the treatment schedule of WT1 peptide vaccine and anti-PD-1 antibody therapies. Arrows and arrowheads represent the time points of the treatments. Asterisks represent the time points of IVIS analysis. (B) Bioluminescence images by IVIS on day 26 after the tumor transplantation. Bioluminescence images for 4 of 15 control mice are those on day 19 since the 4 mice died by day 26. (C) Kaplan-Meier curves for overall survival in each treatment. Asterisks represent the significant difference in the survival (*n* = 15 in each experiment, *P* < .05).

After engraftment of GL261-WT1-luc cells transplanted into the mice was confirmed by IVIS on day 5, a cocktail of WT1_126–134_ killer and WT1_35–52_ helper peptides emulsified with Montanide ISA 51 adjuvant, anti-PD-1 antibody, or the combination of the two therapies was administered as shown in a Figure 2A. Bioluminescence images were obtained weekly for the evaluation of tumor volume by IVIS from days 5 to 54. Tumor shrunk after the treatment and became undetectable on day 26 in 4 of the 15 WT1 peptide vaccine-treated mice and 9 of the 15 anti-PD-1 antibody-treated mice (Figure 2B). On the other hand, tumor shrunk after the treatment and became undetectable on day 26 in all mice treated with the combination therapy of WT1 peptide vaccine and anti-PD-1 antibody, and all but one mice survived for more than 66 days (Figure 2B and C). These results showed that the combination of WT1 peptide vaccine and anti-PD-1 antibody therapies had exerted a synergistic antitumor effect against glioblastoma. In contrast, the control mice all developed tumors and 4 of the 15 control mice had died by day 26 (Figure 2B). Median survival time was 27.5 days (range: 24–39 days), 36.0 days (range: 29–66 days), and 61.0 days (range: 25–66 days) for control, WT1 peptide vaccine-, and anti-PD-1 antibody-treated mice, respectively (Figure 2C). Overall survival rates on day 66 were 13.3% and 46.6% for WT1 peptide vaccine- and anti-PD-1 antibody-treated mice, respectively, whereas all control mice had died by day 39 (Figure 2C). There results showed that this model mouse should be a useful tool for a detailed elucidation of the difference in the immunological response between WT1 peptide vaccine and anti-PD-1 antibody therapies. Furthermore, the distinct treatment schedule of the combination therapy was examined. The combination therapy was started from the day 15, when the implanted tumors became massive (Supplementary Figure 1A). Tumor growth was suppressed by the treatment, and one mice had no detectable tumor on day 26 (Supplementary Figure 1B). These results suggested that the combination therapy could suppress not only tumor initiation but also tumor growth.

### Different Mechanisms in Antitumor Immunity Between WT1 Peptide Vaccine and Anti-PD-1 Antibody Therapies

**Figure 3.**
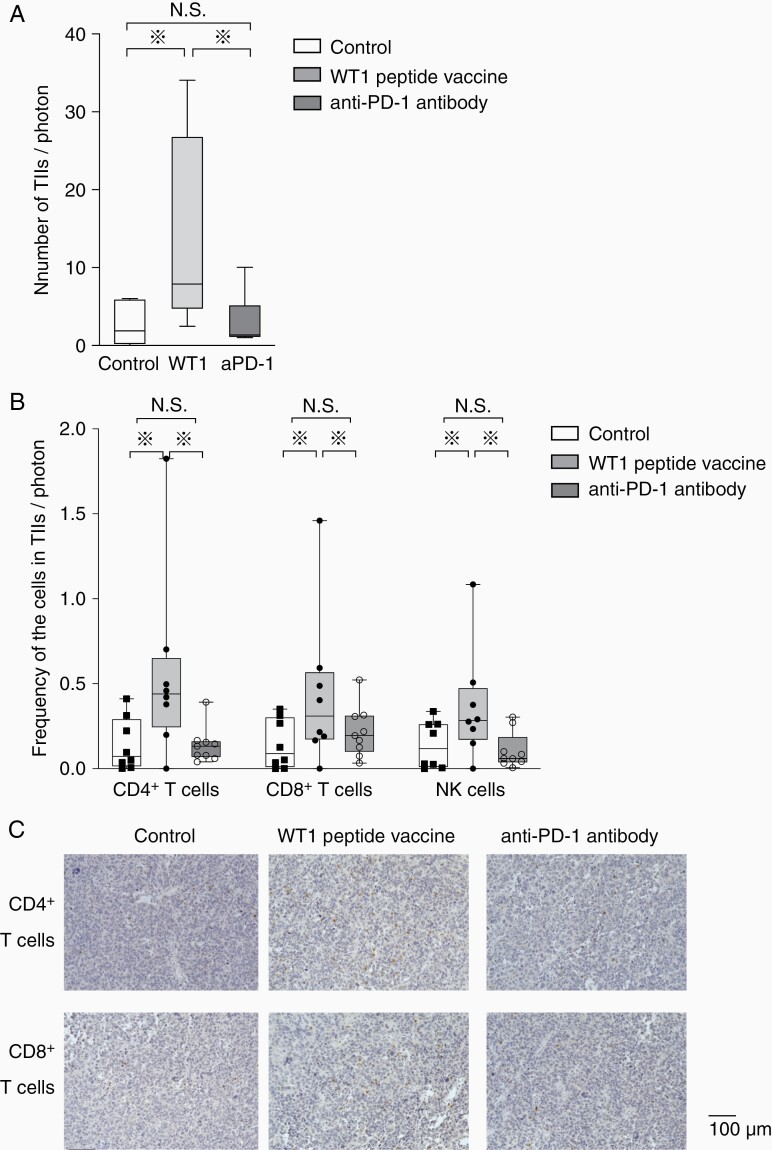
Increased tumor infiltration of CD4^+^ and CD8^+^ T cells and NK cells in WT1 peptide vaccine-treated mice. (A) A box-and-whisker plot of total number of TIIs per tumor-emitting bioluminescence (photons/second) in the control (white column, *n* = 8), WT1 peptide vaccine-treated (gray column, *n* = 8), and anti-PD-1 antibody-treated mice (dark gray column, *n* = 9). (B) Box-and-whisker plots with individual data points of the frequencies of tumor-infiltrating CD4^+^ and CD8^+^ T cell and NK cells per tumor-emitting bioluminescence (photons/second) in the control (white column, *n* = 8), WT1 vaccine-treated (gray column, *n* = 8), and anti-PD-1 antibody-treated mice (dark gray column, *n* = 9). Asterisks represent the significant difference (*P* < .05). (C) Immunohistochemical staining of CD4^+^ (above) and CD8^+^ (below) of resected tumors in mice treated without any therapy, with WT1 peptide vaccine and anti-PD-1 antibody were shown.

TIIs were analyzed for tumors with more than 5.0 × 10^4^ photons of bioluminescence. Since brain tumor masses were too small to resect, the hemisphere of brains with tumors was resected. A cell suspension was then prepared from the hemisphere by using a tumor dissociation kit, and CD45^+^ cells were positively selected as TIIs from the cell suspension. Since the total tumor-emitting bioluminescence reflected the total number of tumor cells, the number of TIIs per one unit of tumor-emitting bioluminescence was measured as an evaluation marker for the infiltration of immune cells into tumors. The number of TIIs per one unit of tumor-emitting bioluminescence was statistically significantly higher in WT1 peptide vaccine-treated mice than anti-PD-1 antibody-treated and control mice (Figure 3A). Importantly, the number of CD4^+^ and CD8^+^ T cells and NK cells per one unit of tumor cell-emitting bioluminescence was also statistically significantly higher in WT1 peptide vaccine-treated mice than anti-PD-1 antibody-treated and control mice (Figure 3B). Consistent with the flow cytometry data, IHC also showed higher infiltration of CD4^+^ and CD8^+^ T cells in WT1 peptide vaccine-treated mice, compared to control and anti-PD-1 antibody-treated mice (Figure 3C). These results clearly demonstrated that WT1 peptide vaccine therapy could induce strong infiltration of both innate and adaptive immune cells into tumors, whereas anti-PD-1 antibody therapy did not have any effect on the induction of infiltration of immune cells into tumors.

**Figure 4.**
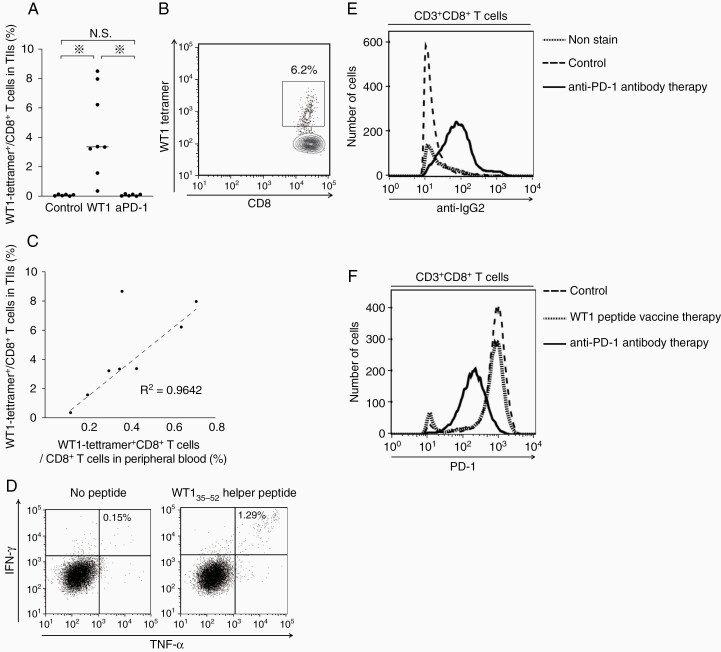
Characterization of TIIs in WT1 peptide vaccine- or anti-PD-1 antibody-treated mice. (A) A bee swarm plot of the frequencies of WT1-tetramer^+^ CD8^+^ T cells among CD8^+^ T cells in TIIs. (B) A representative flow cytometry showing the high frequency of WT1-tetramer^+^ CD8^+^ T cells in TIIs in WT1 peptide vaccine-treated mice. (C) Scatter plots of the frequencies of WT1-tetramer^+^ CD8^+^ T cells in CD8^+^ T cells in TIIs and peripheral blood. Dotted line represents approximate line, and its correlation coefficient (*R*^2^) is 0.9642. (D) A representative flow cytometry of cytokine-producing CD4^+^ T cells in TIIs in WT1 peptide vaccine-treated mice with or without WT1_35–52_ helper peptide stimulation. Intracellular staining of IFN-γ and TNF-α of CD3^+^ CD4^+^ T cells is shown. (E) A representative flow cytometry for the detection of anti-PD-1 antibody (rat IgG2a antibody) on tumor-infiltrating CD3^+^ CD8^+^ T cells in the control, WT1 peptide vaccine-treated, and anti-PD-1 antibody-treated mice. (F) A representative flow cytometry for PD-1 expression in tumor-infiltrating CD3^+^ CD8^+^ T cells in control, WT1 peptide vaccine-treated, and anti-PD-1 antibody-treated mice.

Next, WT1-specific TIIs were examined. The tumor-infiltrating CD3^+^ CD8^+^ T cells were stained with WT1-tetramer antibody. Expectedly, WT1-tetramer^+^ CD8^+^ T cells were detected at high frequencies in WT1 peptide vaccine-treated mice, whereas they were undetectable in anti-PD-1 antibody-treated and control mice (Figure 4A). Representative dots of flow cytometry showed that the frequencies of WT1 tetramer^+^ CD8^+^ T cells were as high as 6.2% in a WT1 peptide vaccine-treated mouse (Figure 4B). Interestingly, there was a positive correlation (*R*^2^ ≑ 0.96) in the frequencies of the WT1 tetramer^+^ CD8^+^ T cells between intratumoral and peripheral blood (Figure 4C). These findings suggest that the frequency of WT1-specific CD8^+^ T cells in peripheral blood can be a prediction marker for the frequencies of the intratumoral WT1-specific CD8^+^ T cells. Furthermore, to examine the frequencies of tumor-infiltrating WT1-specific CD4^+^ T cells, intratumoral CD4^+^ T cells from WT1 peptide vaccine-treated mice were stimulated with WT1_35–52_ helper peptide and their IFN-γ and TNF-α production was measured (Figure 4D). Representative dots of flow cytometry showed that the frequencies of IFN-γ- and TNF-α-producing CD4^+^ T cells were approximately nine times higher in the WT1 peptide-stimulated TIIs than in the nonstimulated mice, showing the infiltration of WT1-specific CD4^+^ T cells into tumor in WT1 peptide vaccine-treated mice. Taken together, these results showed that WT1 peptide vaccine therapy exerted the antitumor effect through the induction of WT1-specific CD8^+^ and CD4^+^ T cells, followed by the infiltration of them into tumors.

In a striking contrast to the TIIs in WT1 peptide vaccine-treated mice, those in anti-PD-1 antibody-treated mice showed different characteristics. Since anti-PD-1 antibody used for the treatment was a rat IgG2a subclass, anti-rat IgG2a antibody was used for the detection of binding of anti-PD-1 antibody on the tumor-infiltrating CD3^+^ CD8^+^ T cells. Expectedly, the majority of the tumor-infiltrating CD8^+^ T cells was stained with anti-rat IgG2a antibody, confirming the binding of anti-PD-1 antibody on the majority of the tumor-infiltrating CD8^+^ T cells (Figure 4E). In contrast, the PD-1 expression on the tumor-infiltrating CD8^+^ T cells was, expectedly, much lower in the anti-PD-1-treated mice than in the WT1 peptide vaccine-treated and control mice (Figure 4F). These results suggested that anti-PD-1 antibody therapy did not increase the infiltration but prevent from exhaustion of tumor-infiltrating CD8^+^ T cells.

Since no mice had detectable tumor on day 26 as shown in Figure 2B, TIIs in mice treated with the combination therapy could not be analyzed under the same condition. Then, the TIIs in mice treated with the combination therapy 15 days after the tumor inoculation as shown in Supplementary Figure 1A were analyzed. WT1-tetramer^+^ CD8^+^ T cells infiltrated into the tumors in all of the six mice treated with the combination therapy (Supplementary Figure 2A). Furthermore, binding of anti-PD-1 antibody on the tumor-infiltrating CD3^+^ CD8^+^ T cells was detected in all of the six mice (Supplementary Figure 2B), and PD-1 expression on the tumor-infiltrating CD8+ T cells was suppressed in four of the six mice (Supplementary Figure 2C). These results suggested that the effect of WT1 peptide vaccine therapy and anti-PD-1 antibody therapy coexisted in mice treated with the combination therapy.

Taken together, our results presented here clearly demonstrate the prominent difference in the mechanisms of antitumor immunity between WT1 peptide vaccine and anti-PD-1 antibody therapies. And their combination led the better survivals than each monotherapy.

## Discussion

The present study was the first report clearly demonstrating the striking different mechanisms in anticancer immunity between TAA peptide-based vaccines such as WT1 peptide vaccine and immune checkpoint inhibitors such as anti-PD-1 antibody for the same tumor under the same microenvironment. Different therapies of WT1 peptide vaccine and anti-PD-1 antibody could be simultaneously performed and both therapies showed clinical efficacy in our mouse glioblastoma model. And, TIIs could be separately analyzed in the same tumors in the WT1peptide vaccine- or anti-PD-1 antibody-treated mice.

Seven steps of the cancer-immunity cycle were proposed to be necessary for the success in cancer immunotherapy: (1) cancer antigen presentation, (2) priming and (3) activation of T cells, (4) trafficking of T cells to tumors, (5) infiltration of T cells into tumor, (6) recognition of cancer cells by T cells, and (7) killing of cancer cells.^8^ WT1 peptide vaccine therapy induced WT1-specific CD4^+^ and CD8^+^ T cells in peripheral blood and infiltrated them into glioblastoma. Furthermore, WT1 peptide vaccine therapy induced strong infiltration of both innate and adaptive immune cells into glioblastoma, resulting in the conversion of the “cold” tumors into “hot” ones. This means that WT1 peptide vaccine therapy can cover steps (2)–(5) of the cancer-immunity cycle. On the other hand, anti-PD-1 antibody therapy did not increase the infiltration of each subset of TIIs but induced low expression of PD-1 molecules on tumor-infiltrating CD8^+^ T cells. This means that anti-PD-1 antibody therapy prevents the exhaustion of tumor-infiltrating CD8^+^ T cells and works specific on the (7) step of the cancer-immunity cycle. Therefore, the combination of WT1 peptide vaccine and anti-PD-1 antibody that covers most of the immunity cycle and showed synergistic antitumor effect. Analysis of late treatment of the combination therapy suggested that the effect of WT1 peptide vaccine therapy and anti-PD-1 antibody therapy coexisted in mice treated with the combination therapy. The PD-1 expression was not suppressed in mice 1 and 2 (Supplementary Figure 2C). The highest tumor growth in these two mice (Supplementary Figure 1B) should be ascribed to no suppression of PD-1 expression on the TIIs of these mice. Furthermore, rechallenge experiment was performed for mice to analyze the immune memory in mice treated with the combination therapy (data not shown). In four tumor-rejected mice, GL261-WT1-luc glioblastoma cells were retransplanted into the contralateral brain hemispheres 125 days after the first transplantation. Bioluminescence images ensured the engraftment of tumors 5 days after the re-transplantation. There was no significant difference in the engraftment assessed by bioluminescence intensity on day 5 after the transplantation between the first transplantation and re-transplantation. Interestingly, all four mice rejected the retransplanted glioblastoma cells without any other therapies and did not have visible tumors at autopsy 33 days after retransplantation. These results indicated that strong immunological memory against glioblastoma cells was induced and maintained in the tumor-rejected mice for more than 125 days. Our results indicated the priority of the combination therapies of WT1 peptide vaccine and anti-PD-1 antibody in cure-oriented cancer immunotherapy.

WT1 peptide vaccine therapy have shown favorable clinical effects against glioblastoma. A phase II clinical trial of WT1 killer peptide vaccination for patients with recurrent glioblastoma succeeded in attaining disease control rates of 57.1%, which was significantly improved compared to those in historical controls.^23^ Notably, one patient had continuously and gradually shrinking tumor for more than 4 years, and two patients are still in stable disease for more than 10 years.^24^ Furthermore, a phase I clinical study of a cocktail vaccine of WT1 killer and helper peptides for patients with recurrent glioblastoma showed a strong induction of WT1-specific CD8^+^ T cells with their long-term maintenance, which might lead to better clinical results.^25,26^ These promising results led to a company-initiated phase I clinical trial for patients with glioblastoma. Seven of 21 patients with glioblastoma survived for ≥1 year, and two of them survived for ≥2 years.^27^ These good results encouraged the company to advance to a phase II clinical trial named “WIZARD 201G Trial” for patients with glioblastoma, which is currently in progress. On the other hand, both CheckMate-143 clinical trial, the first Phase 3 trial randomized multicenter study evaluating nivolumab versus bevacizumab in patients with recurrent glioblastoma, and Checkmate-498, a phase III randomized multicenter study evaluating nivolumab and radiation versus temozolomide and radiation in patients with newly diagnosed glioblastoma, failed to show statistical superiority of nivolumab therapy against glioblastoma.^28,29^ One of the reason of this discrepancy between the results in our mouse model and in clinical settings is that the median percentage of PD-L1-expressing cells in de novo glioblastoma is as low as 2.77%,^30^ whereas the implanted GL261-WT1-luc cells express PD-L1 in the majority of the tumor cells, as shown in Figure 1B and D. High PD-L1expression of the GL261-WT1-luc cells should be ascribed to in vitro selection of such cells during long-term culture. This high PD-L1 expression of the implanted glioblastoma cells might induce strong efficacy of ant-PD-1 therapy in this mouse model.

In conclusion, as shown in our results, WT1 peptide vaccine therapy can convert noninflamed to inflamed states in tumors, therefore, the combination therapy of WT1 peptide vaccine and anti-PD-1 antibody should have a priority for cure-oriented immunotherapy.

## Supplementary Material

vdab091_suppl_Supplementary_Figure_S1

vdab091_suppl_Supplementary_Figure_S2

vdab091_suppl_Supplementary_Figures_Legend

## Notes

**Authorship Statement.** Study conception and design: C.Y., K.T., J.N., and Y.C. Acquisition of data (provided animals, acquired and managed patients, provided facilities, etc.): C.Y., K.T., J.N., H.N., H.M., H.H., and A.K. Analysis and interpretation of data (eg, statistical analysis, biostatistics, and computational analysis): C.Y., K.T., J.N., H.N., R.H., N.K., M.K., Y.H., A.T., Yo.O., Yu.O., H.S., N.K., and H.K. Writing, review, and/or revision of the manuscript: C.Y., J.N., H.S., N.K., and H.K. Administrative, technical, or material support (ie, reporting or organizing data, constructing databases): C.Y., K.T., J.N., and H.H. Study supervision: J.N. All authors reviewed and approved the final version of the manuscript.
