# Supplementary figures and images for: Distinct difference in tumor-infiltrating immune cells between Wilms’ tumor gene 1 peptide vaccine and anti-programmed cell death-1 antibody therapies
Source: Neurooncol Adv. 2021 Jun 29;3(1):vdab091. doi: 10.1093/noajnl/vdab091 (PMC8331049; doi:10.1093/noajnl/vdab091)

Supplementary Figure 1

A

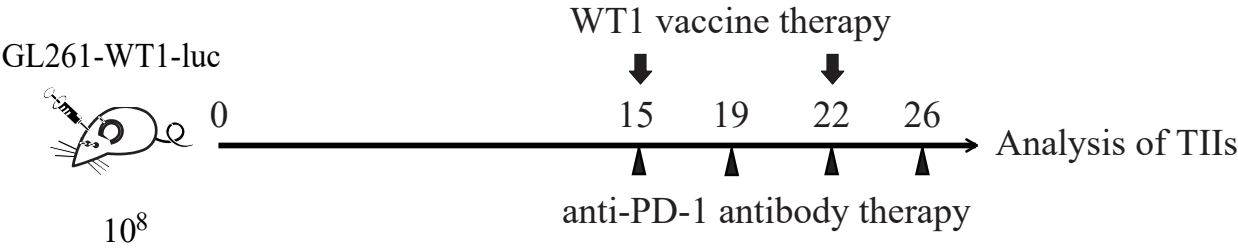

B

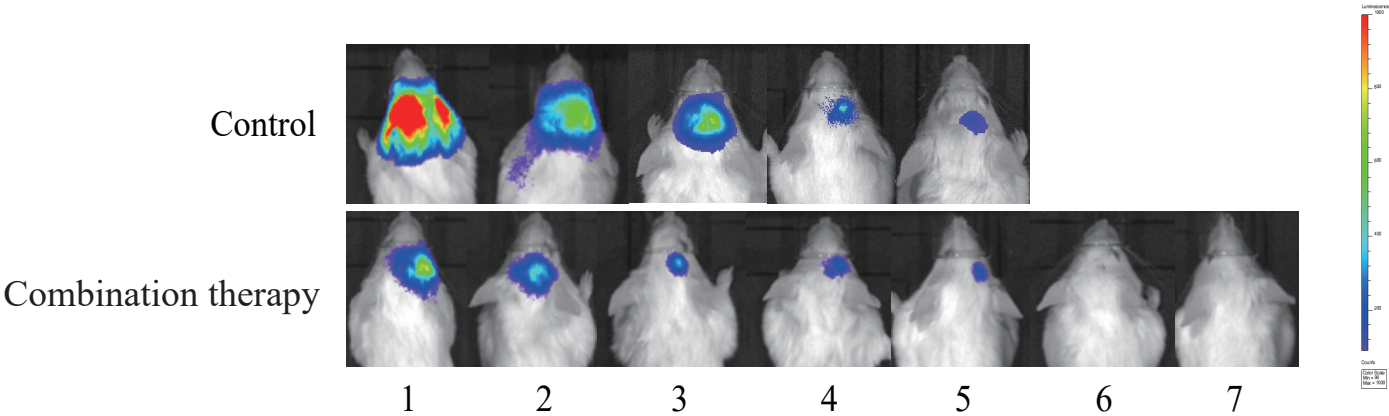

Supplement: vdab091_suppl_Supplementary_Figure_S1 [file vdab091_suppl_supplementary_figure_s1.pdf]

Supplementary Figure 2

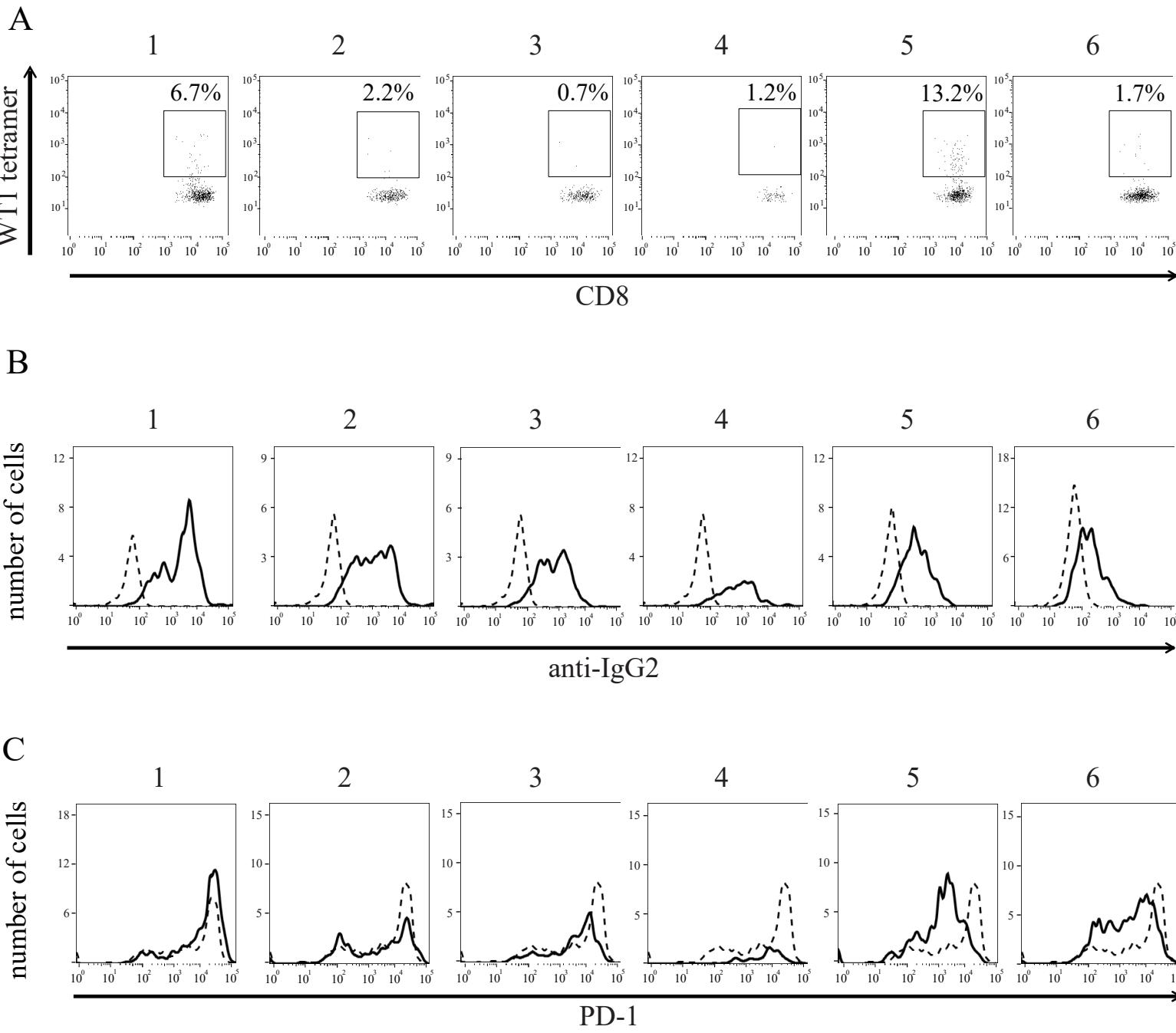

Supplement: vdab091_suppl_Supplementary_Figure_S2 [file vdab091_suppl_supplementary_figure_s2.pdf]
