# Supplementary material for: Distinct difference in tumor-infiltrating immune cells between Wilms’ tumor gene 1 peptide vaccine and anti-programmed cell death-1 antibody therapies
Source: Neurooncol Adv. 2021 Jun 29;3(1):vdab091. doi: 10.1093/noajnl/vdab091 (PMC8331049; doi:10.1093/noajnl/vdab091)
Supplement: vdab091_suppl_Supplementary_Figures_Legend [file vdab091_suppl_supplementary_figures_legend.docx]

**Supplementary figure legends:**

**Supplementary Figure 1. Late treatment of glioblastoma-bearing mice with the combination therapy of WT1 peptide vaccine and anti-PD-1 antibody.**

A. A schema of the treatment schedule of the combination therapy of WT1 peptide vaccine and anti-PD-1 antibody. B. Bioluminescence images by IVIS on day 26 after the tumor transplantation (control: n=5, combination therapy: n=7).

**Supplementary Figure 2. Characterization of TIIs of glioblastoma-bearing mice treated late with the combination therapy of WT1 peptide vaccine and anti-PD-1 antibody.**

A. Flow cytometry of WT1-tetramer^+^ CD8^+^ T cells in tumor-infiltrating CD3^+^CD8^+^ T cells. B. Flow cytometry for the detection of anti-PD-1 antibody (rat IgG2a antibody) bound to tumor-infiltrating CD3^+^CD8^+^ T cells. Solid and dashed lines denote the profiles of tumor-infiltrating CD3^+^CD8^+^ T cells with or without staining with anti-rat IgG2a antibody, respectively. C. Flow cytometry for PD-1 expression on tumor-infiltrating CD3^+^ CD8^+^ T cells. Solid and dashed lines denote the profiles of the combination therapy-treated and control mice, respectively.
